# Awareness, Knowledge, and Attitudes Among Saudi Parents Related to First Aid Practices and Emergency Response to Their Children

**DOI:** 10.7759/cureus.78776

**Published:** 2025-02-09

**Authors:** Faisal A Alghamdi, Arwa A Alghamdi, Israa E Refai, Ali M Ghanam, Reman G Alharbi, Sulaiman F Alhashimi, Shejoon A Alnassri, Ahmed A Alzahrani, Reham N Alsaud, Mokhtar M Shatla

**Affiliations:** 1 College of Medicine, Umm Al-Qura University, Makkah, SAU; 2 Family Medicine, College of Medicine, Umm Al-Qura University, Makkah, SAU; 3 Family Medicine, College of Medicine, Menoufia University, Menoufia, EGY

**Keywords:** allergy and anaphylaxis, awareness, emergency, first aid, hypoglycemia coma, makkah, orthopedic, parents, saudi, trauma

## Abstract

Background

First aid (FA) is vital in caring for children during emergencies, as timely interventions can greatly affect outcomes. Research shows that parents, particularly mothers, are essential in delivering immediate care, which can lower the risk of disability and enhance survival rates for injured children.

Objectives

This study aims to assess Saudi parents' awareness and knowledge of first-aid practices to enhance child safety and health.

Methods

A descriptive cross-sectional study was conducted in Makkah, Saudi Arabia, using an electronic questionnaire distributed to adults aged 18 years and older from November 15 to December 7, 2024. The questionnaire, adapted from a validated tool, assessed sociodemographic data, awareness of FA, attitudes towards FA, and previous training experiences.

Results

Out of 599 participants, 77% demonstrated good knowledge of FA practices. Most respondents were aware of essential first aid measures; however, gaps were noted in specific emergency responses, such as managing hypoglycemic comas and allergic reactions. Factors influencing knowledge included the number of children, prior training, and sources of information.

Conclusions

The study highlights a generally high awareness of first aid among Saudi parents, but specific knowledge gaps necessitate targeted educational interventions. Enhancing first aid training and resources can improve parental preparedness and child safety outcomes.

## Introduction

First aid (FA) is defined by the American Heart Association and the American Red Cross Guidelines in 2010 as "assessments and interventions that can be performed by a bystander or by the victim with minimal or no medical equipment" [1]. FA involves essential skills and knowledge aimed at providing immediate care during emergencies to prevent worsening conditions until professional medical assistance arrives. It is especially critical in situations where medical care may not be immediately accessible such as handling injuries, sudden illnesses, or life-threatening conditions [2].

Parents, particularly mothers, are often the primary caregivers at home and play a crucial role in ensuring their children’s safety. Due to their natural curiosity, children are at a higher risk of accidental injuries or fatalities [3]. Prompt and appropriate parental actions can significantly improve a child’s prognosis, reducing disability and increasing survival rates. Therefore, caregivers must have adequate and appropriate first-aid education and experience [3]. Parents with proper FA training are more confident and effective in managing common childhood emergencies such as choking, burns, or falls [4]. Mothers, who are in close contact with their children during infancy and preschool years face the challenge of creating a safe environment to minimize or prevent harm. Preventing home injuries has become a crucial goal for enhancing children’s health and well-being [5].

Globally, the awareness of FA has been widely studied across various populations [3-7]. However, there is still a notable gap in understanding parents' awareness and preparedness for FA training in Saudi Arabia [6]. This gap is concerning, as timely administration of FA can reduce harm, promote healing, and alleviate suffering following an injury. Improving parents’ FA knowledge and skills is essential to enhancing child health and safety [7].

This study seeks to evaluate parental perspectives on children’s FA to improve health and safety outcomes. As healthcare systems transition toward primary care models, the findings of this research are expected to support healthcare professionals in their roles as health advocates.

## Materials and methods

Study design

Selection and Description of Participants

This descriptive cross-sectional study was conducted in Makkah, Saudi Arabia. Participants were randomly selected by distributing an electronic questionnaire to all adults aged 18 years or older who were willing to participate from November 15 to December 7, 2024. Participants were chosen using random convenience sampling, with the research objectives explained to them through the questionnaire. Participation was voluntary.

Questionnaire Tool

The questionnaire utilized in this study was adapted from the validated tool developed by Al-Johani et al. [3], with modifications and additions made to address the specific objectives of this research. These changes included refining existing questions and adding new ones to better evaluate parents’ knowledge, attitudes, and practices regarding FA for children. The final version of the questionnaire was administered as a self-administered online survey via Google Forms in Arabic, ensuring accessibility and clarity for participants. It is important to note that the mental health status of participants was not specifically assessed or excluded; however, there were no reports of participants having a mental health condition. It consisted of three sections: the first section collected sociodemographic data, including age, sex, marital status, and education level; the second section assessed awareness of first aid and its importance, including prior attendance at FA courses and participants’ self-confidence in handling emergencies; and the third section focused on attitudes toward providing FA to children in common and life-threatening emergencies. The modifications ensured the questionnaire was comprehensive and aligned with evidence-based medical guidelines to enhance its relevance to the study objectives.

Sample Size Calculation

Calculator.net was used to determine the minimum number of samples required for this study. The population of adult residents in Makkah is approximately 5 million, as reported by the General Authority for Statistics in Saudi Arabia [8]. With a confidence interval level of 98%, an expected frequency percentage of 50%, and a design effect of 1, the sample size was calculated to be a minimum of 543 participants.

Ethical approval

This study received approval from the Biomedical Research Ethics Committees of Umm Al-Qura University, with the ethical approval number HAPO-02-K-012-2024-10-2301, and was performed according to the Declaration of Helsinki.

Informed Consent

The questionnaire started with an explanation of the study's objectives to emphasize that participation in the research was optional. Their names were not collected, and the participants' identities were kept private and anonymous. All participants electronically consented to the study.

Statistical analysis

The statistical analysis for the study data was performed using SPSS version 26 (IBM Corp., Armonk, NY, US). Socio-demographic data were summarized using descriptive statistics, including frequencies and percentages for categorical variables (e.g., age, gender, education, and marital status). Knowledge and awareness were evaluated by scoring correct responses as 1 point, with an overall score calculated for each participant. A cutoff of 60% was used to classify knowledge levels into "poor" and "good." Associations between socio-demographic factors and overall knowledge levels were analyzed using the Pearson chi-square test (χ²) or Fisher's exact test when appropriate, with significance set at p<0.05. Additionally, the relationship between training experience, perception, and overall knowledge was assessed using the chi-square or Fisher's exact test.

## Results

Table 1 shows the socio-demographic characteristics of the study parents in Makkah, Saudi Arabia (n=599). The most frequent age group was 41-50 years (228; 38.1%), followed by 31-40 years (159; 26.5%), while only 92 (15.4%) were aged 18-30. Females constitute the majority of the respondents 457 (76.3%), with males being 140 (23.7%). Most participants have attained a university-level education 338 (56.4%), while 140 (23.4%) have secondary education or below, and a smaller proportion hold postgraduate qualifications 63 (10.5%). Regarding monthly income, 269 (44.9%) reported 10,000 SR, while 185 (30.9%) reported less than 5,000 SR. An exact 461 (77%) parents had an overall good knowledge and awareness level while 138 (23%) had a poor knowledge level about FA practices and emergency response to their children. As for sources of parents' information about FA practices, the most reported were social media (384; 64.3%), family/friends (212; 35.5%), training courses (192; 32.2%), and health care staff (167; 28%).

**Table 1:** Factors associated with parent's overall knowledge and awareness level P: Pearson X2 test; ^: Exact probability test; * P < 0.05 (significant)

Factors	Overall knowledge and awareness level				p-value
	Poor		Good		
	No	%	No	%	
Age in years					0.811
18-30	24	26.1%	68	73.9%	
31-40	35	22.0%	124	78.0%	
41-50	54	23.7%	174	76.3%	
> 50	25	20.8%	95	79.2%	
Gender					0.602
Male	35	24.6%	107	75.4%	
Female	103	22.5%	354	77.5%	
Educational level					0.176
Secondary / below	41	29.3%	99	70.7%	
Diploma	15	25.9%	43	74.1%	
University graduate	70	20.7%	268	79.3%	
Post-graduate	12	19.0%	51	81.0%	
Marital status					0.276
Married	130	23.6%	421	76.4%	
Divorced / widow	8	16.7%	40	83.3%	
Number of children					0.017*
1-3	59	22.2%	207	77.8%	
4-6	55	20.5%	213	79.5%	
> 6	24	36.9%	41	63.1%	
Have you previously attended any first-aid training courses?					0.004*
Yes	48	17.6%	225	82.4%	
No	90	27.6%	236	72.4%	
Did the course include practical application?					0.042*^
Yes	36	15.6%	195	84.4%	
No	12	28.6%	30	71.4%	
Source of information					0.001*^
None	19	90.5%	2	9.5%	
Social media	82	21.4%	302	78.6%	
Doctors and nurses	29	17.4%	138	82.6%	
Family or friends	43	20.3%	169	79.7%	
Training courses	29	15.1%	163	84.9%	
Others	2	10.5%	17	89.5%	

Table 2 provides the FA training history and experience among parents in Makkah, Saudi Arabia. The vast majority (544; 90.8%) knew the ambulance number and almost all participants (595; 99.3%) believed that practicing FA is important. Less than half (273; 45.6%) have attended a first-aid training course. Among those who attended, 231 (84.6%) received practical training. Most parents (589; 98.3%) feel that FA courses are very important; only 159 (26.5%) feel highly confident in dealing with children's emergencies, with 342 (57.1%) having some confidence and 98 (16.4%) lacking confidence. A large majority (558; 93.2%) would attend an FA training course if invited.

**Table 2:** Relationship between training experience, perception, and overall knowledge and awareness of first aid among parents in Saudi Arabia P: Pearson X2 test; ^: Exact probability test; * P < 0.05 (significant)

Training experience and perception	Overall knowledge and awareness level				p-value
	Poor		Good		
	No	%	No	%	
Do you know the ambulance number in the Kingdom of Saudi Arabia?					0.001*
Yes	115	83.3%	429	93.1%	
No	23	16.7%	32	6.9%	
Do you think it is important to practice first aid?					0.001*^
Yes	134	97.1%	461	100.0%	
No	4	2.9%	0	0.0%	
Do you feel that first aid courses are of great importance?					0.001*^
Yes	131	94.9%	458	99.3%	
No	7	5.1%	3	0.7%	
Do you feel confident in dealing with your children's emergencies?					0.001*
No confidence	39	28.3%	59	12.8%	
Somewhat confidence	69	50.0%	273	59.2%	
High confidence	30	21.7%	129	28.0%	
If you were invited to attend a first aid training course, would you attend?					0.001*
Yes	118	85.5%	440	95.4%	
No	20	14.5%	21	4.6%	

Regarding Saudi parents' awareness and knowledge of FA practices and emergency responses for their children (Table 3), most parents (578; 96.5%) had heard about FA for injuries. In the case of epileptic seizures, only 342 (57.1%) correctly identified supporting the child’s head and placing them in the recovery position as the appropriate response. For fainting due to low blood sugar, only 89 (14.9%) correctly know they should call an ambulance. Asthma attacks had a strong correct response rate, with 525 (87.6%) identifying the use of an inhaler as the appropriate action. Regarding allergy attacks, 294 (49.1%) correctly selected transporting the child directly to the hospital, though 162 (27%) chose to administer a sensitivity injection. For bleeding wounds, 432 (72.1%) accurately opted for covering the wound and applying pressure. Similarly, 397 (66.3%) correctly chose washing a burn area with cold water. In the case of suffocation, 390 (65.1%) correctly identified chest-abdominal compressions, with some 144 (24%) erroneously opting for hitting the back. For electric shocks, 534 (89.1%) knew to avoid direct contact by removing the source of electricity safely. Nosebleed management showed a correct response of 287 (47.9%), but a concerning 221 (36.9%) incorrectly suggested raising the head backward. Finally, 561 (93.7%) demonstrated proper handling of suspected fractures by immobilizing the affected part and seeking hospital care.

**Table 3:** Awareness and knowledge among Saudi parents related to first aid practices and emergency response to their children $: the correct answer

Knowledge and awareness		No	%
Have you heard about first aid for injuries?	Yes	578	96.5%
	No	21	3.5%
The correct behavior in the event of epileptic seizures?	Support the child's head and place him in the recovery position until the seizure ends ^$^	342	57.1%
	Try to reduce the child's movement by stabilizing him	97	16.2%
	Giving the child medication for epilepsy	57	9.5%
	Trying to feed or water the child	2	0.3%
	I don't know	101	16.9%
The correct behavior in the event of fainting due to low blood sugar?	Call the ambulance ^$^	89	14.9%
	Feed the child some food that contains sugar	460	76.8%
	Sprinkle water on the child's face	13	2.2%
	I don't know	37	6.2%
The correct behavior in case of asthma attacks?	Give the child an asthma inhaler ^$^	525	87.6%
	Waiting for the attack to end	15	2.5%
	Make the child do some physical activities	3	0.5%
	I don't know	56	9.3%
The correct behavior in the event of allergy attacks?	Transport the child directly to the hospital ^$^	294	49.1%
	Give the sensitivity injection directly	162	27.0%
	Use some painkillers	68	11.4%
	I don't know	75	12.5%
If your child is exposed to a bleeding wound, what is the correct course of action in this case?	Cover the wound and apply pressure to the site of the injury ^$^	432	72.1%
	Go directly to the hospital	108	18.0%
	Wash the wound with water	27	4.5%
	Place coffee, or honey on the wound site	17	2.8%
	Apply natural herbs to the wound	1	0.2%
	I don't know	14	2.3%
If your child is exposed to burns, what is the correct course of action in this case?	Wash the area with cold water ^$^	397	66.3%
	Cover the burned area	68	11.4%
	Place nature's herbs on the burn	19	3.2%
	Apply toothpaste or tomato paste to the burned area	67	11.2%
	I don't know	48	8.0%
If your child suffocates, what is the correct behavior in this case?	Do chest-abdominal compressions to try to dislodge the body ^$^	390	65.1%
	Hit the child on the back to try to get the body out	144	24.0%
	Trying to remove the foreign body with my hand	35	5.8%
	Give the child an amount of water to swallow the body	3	0.5%
	I don’t know	27	4.5%
If your child is exposed to an electric shock, what is the correct behavior in this case?	Try to remove it from the electricity source without touching it ^$^	534	89.1%
	Call an ambulance	38	6.3%
	Spray the child with water to stop the electricity	2	0.3%
	I don't know	25	4.2%
If your child experiences a nosebleed (epistasis), what is the correct course of action in this case?	Press the nose and wait for the bleeding to stop ^$^	287	47.9%
	Raise the child's head back	221	36.9%
	Block the nostrils by placing tissues	42	7.0%
	Leave the bleeding without intervention	16	2.7%
	I don't know	33	5.5%
The correct behavior if your child falls and is suspected of having suffered a broken limb?	Immobilize the affected part, do not move it, and transport it to the hospital ^$^	561	93.7%
	Apply warm compresses to the affected part	10	1.7%
	Give some painkillers	5	0.8%
	Move the affected part	1	0.2%
	I don't know	22	3.7%

The overall knowledge and awareness of study parents about FA practices and emergency response to their children. An exact 461 (77%) parents had an overall good knowledge and awareness level while 138 (23%) had a poor knowledge level. As for sources of parents' information about FA practices, the most reported were social media (384; 64.3%), family/friends (212; 35.5%), training courses (192; 32.2%), and health care staff (167; 28%).

Table 4 presents the factors associated with the overall knowledge and awareness level of parents. There is a significant association between the number of children and the level of knowledge as parents with fewer than 3 children (207 or 77.8% with good knowledge) and with 4-6 children (213 or 79.5% with good knowledge) exhibited higher levels of knowledge compared to those with more than 6 children (41 or 63.1% with good knowledge) (p = 0.017). Also, attending first-aid training courses is significantly related to knowledge level, as those who attended such courses had a higher percentage of good knowledge (225; 82.4%) compared to those who did not attend (236 or 72.4% with good knowledge) (p = 0.004). The inclusion of practical application in first-aid training shows a significant effect on knowledge where parents who received practical training displayed a higher percentage of good knowledge (195 or 84.4%) compared to those who did not (30 or 71.4% with good knowledge) (p = 0.042). The source of information is strongly associated with knowledge level where parents who received information through social media, doctors, nurses, family, or training courses demonstrated significantly higher percentages of good knowledge: 302 (78.6%), 138 (82.6%), 169 (79.7%), and 163 (84.9%), respectively. In contrast, parents who relied on no source of information had a very low percentage of good knowledge (2; 9.5%) (p = 0.001). Age, gender, and all other remaining factors showed insignificant relation with parents' overall knowledge level.

**Table 4:** Factors associated with parent's overall knowledge and awareness level P: Pearson X2 test; ^: Exact probability test; * P < 0.05 (significant)

Factors	Overall knowledge and awareness level				p-value
	Poor		Good		
	No	%	No	%	
Age in years					0.811
18-30	24	26.1%	68	73.9%	
31-40	35	22.0%	124	78.0%	
41-50	54	23.7%	174	76.3%	
> 50	25	20.8%	95	79.2%	
Gender					0.602
Male	35	24.6%	107	75.4%	
Female	103	22.5%	354	77.5%	
Educational level					0.176
Secondary / below	41	29.3%	99	70.7%	
Diploma	15	25.9%	43	74.1%	
University graduate	70	20.7%	268	79.3%	
Post-graduate	12	19.0%	51	81.0%	
Marital status					0.276
Married	130	23.6%	421	76.4%	
Divorced / widow	8	16.7%	40	83.3%	
Number of children					0.017*
1-3	59	22.2%	207	77.8%	
4-6	55	20.5%	213	79.5%	
> 6	24	36.9%	41	63.1%	
Have you previously attended any first-aid training courses?					0.004*
Yes	48	17.6%	225	82.4%	
No	90	27.6%	236	72.4%	
Did the course include practical application?					0.042*^
Yes	36	15.6%	195	84.4%	
No	12	28.6%	30	71.4%	
Source of information					0.001*^
None	19	90.5%	2	9.5%	
Social media	82	21.4%	302	78.6%	
Doctors and nurses	29	17.4%	138	82.6%	
Family or friends	43	20.3%	169	79.7%	
Training courses	29	15.1%	163	84.9%	
Others	2	10.5%	17	89.5%	

Table 5 presents the relationship between training experience, perception, and overall knowledge and awareness of first aid among parents in Saudi Arabia. Parents with good overall knowledge and awareness showed significantly higher familiarity with the ambulance number (429; 93.1% vs. 115; 83.3%), (p = 0.001) and were more likely to recognize the importance of practicing first aid (461; 100.0% vs. 134; 97.1%), (p =0.001). Similarly, the importance of first aid courses was more strongly recognized by those with good knowledge (458; 99.3% vs. 131; 94.9%), (p =0.001). Also, parents with good knowledge exhibited higher levels of confidence in dealing with children's emergencies, with 28.0% reporting high confidence compared to 30 (21.7%) in the poor knowledge group (p=0.001). Additionally, willingness to attend a first aid training course was higher among parents with good knowledge (440; 95.4%) vs. 118; 85.5%), (p =0.001).

**Table 5:** Relationship between training experience, perception, and overall knowledge and awareness of first aid among parents in Saudi Arabia P: Pearson X2 test; ^: Exact probability test; * P < 0.05 (significant)

Training experience and perception	Overall knowledge and awareness level				p-value
	Poor		Good		
	No	%	No	%	
Do you know the ambulance number in the Kingdom of Saudi Arabia?					0.001*
Yes	115	83.3%	429	93.1%	
No	23	16.7%	32	6.9%	
Do you think it is important to practice first aid?					0.001*^
Yes	134	97.1%	461	100.0%	
No	4	2.9%	0	0.0%	
Do you feel that first aid courses are of great importance?					0.001*^
Yes	131	94.9%	458	99.3%	
No	7	5.1%	3	0.7%	
Do you feel confident in dealing with your children's emergencies?					0.001*
No confidence	39	28.3%	59	12.8%	
Somewhat confidence	69	50.0%	273	59.2%	
High confidence	30	21.7%	129	28.0%	
If you were invited to attend a first aid training course, would you attend?					0.001*
Yes	118	85.5%	440	95.4%	
No	20	14.5%	21	4.6%	

## Discussion

In many cases, accidents involving children at home can be prevented or reduced by parents who are prepared to take early action when an incident occurs [7]. Research indicates that parents with higher levels of education and those who have undergone first-aid training are generally more informed about effective first-aid administration, which can help prevent accidents or reduce their severity [3,6]. Due to the lack of clear insights into the awareness and knowledge of first aid measures among parents in many western regions of Saudi Arabia, this study was conducted to evaluate parental awareness of first aid (FA) for children [3-7]. The aim of this study is to enhance child health and safety overall.

The study assessed parents' overall knowledge and awareness of first aid practices and emergency response for their children. Exactly 461 parents (77%) demonstrated a good level of knowledge and awareness while 138 parents (23%) exhibited a poor knowledge level.

The most frequent age group among study participants was 41-50 years (38.1%), with females constituting the majority of respondents (76.3%). Most participants attained a university-level education (56.4%), and 44.9% reported a monthly income of 10,000 SR or more. Additionally, a significant majority of the respondents were married (92%), and nearly equal proportions reported having 1-3 children (44.4%) and 4-6 children (44.7%).

In the current study, the vast majority of parents (96.5%) reported being aware of first aid for injuries. This statistic is comparable to a survey conducted in Al-Medinah, Saudi Arabia, where awareness was reported at 97.2% [3]. On the other hand, a study involving rural mothers in Egypt found that 26.6% were unfamiliar with the concept of first aid [9]. Additionally, research conducted in India indicated that 65.7% of participants had not been exposed to first-aid information before the study [10]. These contrasting figures highlight a significant disparity in first aid awareness across different regions. While urban populations appear to have a high level of awareness, rural communities may lack access to essential health education. This raises important questions about the effectiveness of health communication strategies and the need for targeted interventions to bridge these gaps.

In the current study, only 57.1% of parents correctly identified the appropriate responses to epileptic seizures, directly supporting the child's head and placing them in the recovery position. This result is significantly different from one from India, where only 6.4% [10] of participants demonstrated knowledge of managing seizures effectively. Additionally, the same previous survey in Al-Medinah City found that 55.4% [3] of parents knew that epileptic seizures usually begin and end spontaneously without the need for special precautions. A study in Kingston, Jamaica, revealed that 54% of participants knew of appropriate actions to take during a seizure [11]. These disparities highlight significant gaps in first aid knowledge across different populations, suggesting a need for targeted educational interventions to improve awareness and response strategies for seizure management.

The current study found that only 14.9% of participants were aware that the appropriate response to a hypoglycemic coma is to call an ambulance and transport the child to the hospital. This indicates a concerning level of knowledge among respondents. In contrast, the study in Al-Madinah City found that mothers demonstrated satisfactory knowledge when handling emergency situations related to diabetes. This study noted that nearly two-thirds of mothers could identify the symptoms of hyperglycemia and more than half could recognize the symptoms of hypoglycemia [3]. This suggests a significantly higher level of awareness and preparedness in Al-Madinah compared to the current study. Thus, the disparity in awareness underscores a critical gap in education that could endanger children's health during emergencies. While mothers in Al-Madinah demonstrated proactive learning about diabetes management, the current study reveals a pressing need for targeted educational interventions. Enhancing understanding of diabetes-related emergencies can improve parental readiness and highlight the importance of community health programs to increase awareness of first aid and emergency responses.

Moreover, in the current study, the response rate for recognizing the appropriate action during asthma attacks was notably high, with 87.6% of participants correctly identifying using an inhaler. Conversely, a study conducted in China revealed that over half (51.34%) of parents scored ≤ 18 on the knowledge, attitudes, and practices (KAP) questionnaire, indicating a significant deficiency in parental knowledge regarding asthma management [12]. In contrast, participants in Riyadh demonstrated a higher level of understanding, with 78.4% exhibiting moderate knowledge of asthma-related aspects [13]. Regarding allergy attacks, 49.1% of participants correctly selected transporting the child directly to the hospital, although 27% chose to administer a sensitivity injection. The findings highlight a significant variation in parental knowledge regarding asthma and allergy management. While the high response rate for recognizing inhaler use is encouraging, the contrasting data from China emphasizes the need for improved educational resources for parents. The moderate understanding observed in Riyadh suggests a positive trend, yet it also points to the necessity for ongoing training. The mixed responses concerning allergy treatment indicate potential confusion among parents, reinforcing the importance of targeted interventions to enhance overall awareness and preparedness in managing respiratory emergencies.

Furthermore, in the presented study, the response rate for recognizing the appropriate action during allergy attacks was average, with 49.1% of participants correctly identifying the need to transport a child directly to the hospital. A study conducted in the Veneto region of Italy revealed poor parental preparedness and reluctance to use adrenaline auto-injectors (AAIs), despite a high to moderate self-rated knowledge among parents. While most parents (77%) reported carrying an AAI, only 20% utilized it in the event of a severe reaction. Barriers to AAI use included fear of making mistakes and concerns about possible side effects. Additionally, poor knowledge of correct AAI usage and incorrect assessment of symptoms were reported, though less frequently [14]. In a study conducted in the western region of Saudi Arabia, which overlaps with the demographic of our current study, concerning gaps in knowledge about anaphylaxis among the general population were revealed. Specifically, only 18.4% recognized epinephrine as the first-line treatment, and only 43.1% understood the necessity of visiting the emergency room after administering epinephrine. These findings highlight the urgent need for improved education and awareness regarding anaphylaxis management among parents and the general public [15].

Additionally, the response rate in the presented study for recognizing the appropriate action for bleeding wounds was notably high, with 72.1% of participants accurately opting for covering the wound and applying pressure. Conversely, the same study conducted in Al-Madinah City revealed that only 21% of parents knew that the first step in caring for a bleeding wound was compression at the bleeding site [3]. In a study conducted in the Makkah region among the general population, less than 20% of participants provided correct responses regarding applying pressure on the source of the bleeding to care for wounds [16]. Additionally, a study done in El-Beheira Governorate, Egypt, found that only 14.2% of respondents took the correct action regarding cut wounds [17]. The findings from these studies reveal a concerning gap in knowledge regarding first aid for bleeding wounds across different populations. These disparities suggest that many individuals may be unprepared to respond effectively in emergencies, which could lead to increased risks of complications or poor outcomes.

In the current study, 66.3% of participants correctly chose the appropriate response to wash a burn area with cold water. In the same survey conducted in Al-Medinah, 73.1% of parents knew that a person with burned clothes should be covered with a blanket, and 45.9% understood that the burn area should be kept under cold water [3]. In Riyadh, however, more than half of parents (56.5%) exhibited low awareness regarding burn first aid, with only 5.8% demonstrating a high level of awareness [6]. In India, the correct response rate was only 26.4%, indicating a limited understanding of burn care [10]. In the Al-Baha region, the majority of participants (73.6%) also had inadequate knowledge of first aid for burns [18]. These findings demonstrate a notable level of awareness regarding burn first aid among participants in our study. In contrast, significantly lower awareness levels observed in other regions highlight a critical gap in knowledge. This disparity underscores the pressing need for targeted educational initiatives to enhance burn care understanding across diverse populations.

Sixty-five point one percent (65.1%) of this study found that parents correctly identified chest-abdominal compressions as the appropriate response to suffocation. In the same previous study conducted in Al Madinah, only 12.8% of participants knew the correct choking actions [3]. Conversely, a study in Riyadh indicated that 38% of parents had low awareness regarding first aid [6]. Additionally, a survey in Nottingham assessed parents' knowledge and confidence related to infant first aid, revealing that 75% of participants knew the correct procedure for choking [17]. According to these findings from previous studies, parents' knowledge of how to react when suffocation occurs should be enhanced and evaluated.

Only 47.9% of current survey participants provided the correct response for managing nosebleeds while 36.9% incorrectly suggested raising the head back. In the Al-Medinah study, just 27.9% of parents demonstrated good knowledge of nosebleed management [3]. Similarly, a survey conducted in Al-Taif City revealed that about two-thirds (67.5%) of parents had a moderate level of knowledge. In comparison, approximately one-third (30.9%) showed a good understanding of first aid for epistaxis [19]. Moreover, in a study conducted in Arar, Saudi Arabia, only 40.4% of parents could correctly identify all necessary first-aid steps for managing nosebleeds [20]. Additionally, a study at the University of Sains Malaysia found that only 21.7% had a high level of knowledge and good practice regarding the first aid management of epistaxis [21]. This suggests that the level of awareness and understanding regarding nosebleed management should be raised, emphasizing the need for targeted educational efforts to improve first-aid knowledge in the community.

In the current study, 93.7% of participants demonstrated proper handling of suspected fractures by immobilizing the affected area and seeking hospital care. In the Al-Medinah study, slightly more than half of the participants (51.8%) understood that in the case of a suspected hand fracture, a hand slap should be applied before transferring the patient to the hospital [3]. A study conducted at pediatric outpatient clinics at Tanta University Hospital in Egypt found that mothers had a knowledge level of 75% regarding first aid for wounds and fractures [22], which is similar to our results. In Buraidah City, Saudi Arabia, the correct response rate for first aid in fractures was 41.5% [5]. This suggests that parents' knowledge of how to handle suspected fractures should be raised.

To improve these statistics, targeted educational programs and workshops aimed at increasing parents' knowledge of first-aid practices are essential. Enhanced awareness can significantly impact children's safety and health outcomes, equipping parents with the skills necessary to respond effectively in emergencies. Moreover, addressing specific gaps identified in the study, such as managing seizures, hypoglycemic comas, and allergic reactions, should be a priority in curriculum development. By empowering parents with comprehensive first-aid knowledge, we can improve emergency responses and ultimately enhance the overall health and safety of children in the community. The pressing need for these educational interventions cannot be overstated, as they play a crucial role in preparing parents to handle emergencies effectively.

In the current study, 99.3% of participants acknowledged the significance of practicing first aid, aligning with findings from earlier studies in Al Madinah [3], Egypt [9], and India [10] that yielded similar results. Most participants expressed a desire to receive training, underscoring the need for authorities to implement additional training programs.

In the present study, nearly half of the parents indicated that they had participated in first-aid training courses, which typically included practical sessions. In a study conducted in Al Madinah [3], only 22% of mothers reported having received formal training in pediatric first aid.

In the present study, the most frequently cited source of information was social media, accounting for 64.3% of responses. Similarly, in Egypt, 56.1% of rural mothers indicated that their knowledge came from TV and radio [9]. In India, social media constituted 45.8% of knowledge sources. A prior study conducted in Al Madinah, Saudi Arabia, also identified social media as the primary source of first aid information for parents [3]. Also in Al-Madinah, 59% of responses agreed that social media is a primary source of information compared to other sources. These findings emphasize the significant role of social media in disseminating first aid information to the general public, highlighting the need to ensure the quality of information shared on these platforms.

The current study explored various factors associated with FA knowledge and found that parents with higher education levels were more likely to possess better FA knowledge than those with lower education. Additionally, parents with a moderate number of children (4-6) demonstrated greater knowledge compared to those with a larger number of children (more than 6). Parents who identified training courses as their primary source of information were more knowledgeable than those who relied on information from family or friends. This underscores the vital role of training programs in imparting first aid knowledge. 

Furthermore, parents who had previously attended first aid training reported higher knowledge levels compared to those who had not. Therefore, participating in first aid training courses is crucial for enhancing knowledge and practice in this area. Parents who knew the ambulance number had better first aid knowledge than those who did not; however, due to the cross-sectional design of the study, it is possible that greater knowledge led to awareness of the ambulance number rather than the other way around.

In a related study conducted in Egypt, mothers who were younger, had university or postgraduate education, worked in health-related occupations, had higher socioeconomic status, and had attended first aid training courses scored the highest in knowledge [9]. Similarly, in Singapore, it was reported that mothers with higher education levels were more likely to achieve greater knowledge and practice scores concerning first aid for childhood injuries [23]. In Turkey, a study by Tomruk found that mothers who were healthcare professionals, had graduated from university, completed a first aid course, or held a first aid certificate showed superior first aid knowledge [24].

The study possesses several strengths. Starting by including a good sample size of 599 participants enhances the reliability of the findings and ensures a diverse demographic representation across various age groups, genders, educational levels, and family sizes. Furthermore, it employs a questionnaire adapted from a validated tool, ensuring effective measurement of relevance. The study also highlights the significance of practical application in first aid training, demonstrating its correlation with improved parental knowledge.

However, this research has certain limitations that should be acknowledged. First, the cross-sectional design restricts the ability to establish causal relationships between knowledge levels and training experiences. Second, the reliance on self-reported data may introduce response bias, as parents might overestimate their knowledge. Finally, the study was conducted in Makkah, Saudi Arabia, which may limit the generalizability of findings to other regions.

Future research should explore longitudinal designs to assess changes in first-aid knowledge following training interventions and to evaluate the effectiveness of various educational approaches. Additionally, the development of standardized first aid training programs tailored specifically for parents, combined with public health campaigns that disseminate accurate information through social media, could significantly improve child safety outcomes. Expanding the scope of the study to include diverse regions would provide a more comprehensive understanding of parental first aid awareness and needs across the country.

## Conclusions

This study evaluated the awareness, knowledge, and attitudes of Saudi parents in Makkah regarding first aid and emergency responses for children. While most parents are aware of first aid, there are significant knowledge gaps in handling emergencies like hypoglycemic coma, seizures, and nosebleeds. The study highlights the importance of first-aid training in improving parental preparedness and found that parents with prior training demonstrated better knowledge. Social media was identified as the main source of first aid information, emphasizing the need for accurate, evidence-based content on these platforms.
